# Primary biliary cirrhosis and osteoporosis: a bidirectional two-sample Mendelian randomization study

**DOI:** 10.3389/fimmu.2023.1269069

**Published:** 2023-12-14

**Authors:** Diqian Zhao, Guobi Li, Wenzhe Bai, Jiawen Teng, Bing Yan, Cong Han

**Affiliations:** ^1^ The First Clinical Medical School, Shandong University of Traditional Chinese Medicine, Jinan, China; ^2^ Department of Pediatric Orthopedics, Affiliated Hospital of Shandong Traditional Chinese Medicine University, Jinan, China; ^3^ Department of Micro Orthopedics, Affiliated Hospital of Shandong Traditional Chinese Medicine University, Jinan, China; ^4^ Department of Joint Oncology Orthopedics, Affiliated Hospital of Shandong Traditional Chinese Medicine University, Jinan, China; ^5^ Nephropathy Department, Affiliated Hospital of Shandong University of Traditional Chinese Medicine, Jinan, China

**Keywords:** primary biliary cirrhosis, osteoporosis, Mendelian randomization study, genome-wide association studies, causal relationship

## Abstract

**Background:**

Observational studies have identified a heightened risk of osteoporosis and fractures in patients with primary biliary cholangitis (PBC). However, conclusive evidence establishing a causal relationship between the two, and a clear mechanism explaining this association, remains elusive.

**Methods:**

We conducted a bidirectional two-sample Mendelian randomization (MR) analysis to investigate the causal relationship between PBC and osteoporosis. This analysis utilized five MR methods: inverse-variance weighted (IVW), MR-Egger, weighted median, weighted mode, and simple mode. Sensitivity analyses were performed, employing various models and testing methods, to assess the impact of heterogeneity and pleiotropy on the results and to confirm their robustness.

**Results:**

A causal relationship between PBC and osteoporosis risk was established through IVW analysis (OR: 1.049, 95%CI: 1.017–1.082, *P*=0.002). Three other MR analyses corroborated these findings. Conversely, osteoporosis was not found to causally affect PBC risk, as evidenced by IVW analysis (OR: 0.941, 95%CI: 0.783–1.129, *P*=0.511). Across all MR analyses, no heterogeneity or horizontal pleiotropy was detected among the instrumental variables (IVs). Furthermore, the leave-one-out analysis indicated that no single SNP disproportionately influenced the results, affirming the reliability of the bidirectional MR findings.

**Conclusion:**

This study establishes a positive causal relationship between PBC and the risk of osteoporosis, while no definitive causal link was found from osteoporosis to PBC. These findings offer new insights and guidance for managing bone health in PBC patients.

## 1 Introduction

Primary biliary cholangitis (PBC), formerly known as primary biliary cirrhosis, is an autoimmune disease characterized by chronic inflammatory damage to the liver. The precise etiology and mechanisms underlying PBC are not yet fully understood. Its hallmark is chronic non-suppurative destructive cholangitis, primarily affecting the interlobular and septal bile ducts, resulting in periductal inflammation and necrosis (1). The incidence and prevalence of PBC show significant variation across different regions and over time, influenced by genetic, environmental, and socio-economic factors. According to recent data (2), the incidence of PBC increased until 2000, then stabilized in North America and Europe, but continued to grow in the Asia-Pacific region. The global prevalence of PBC is on the rise, with North America reporting the highest rate of 21.81 per 100,000 individuals, followed by Europe at 14.59 per 100,000, and the Asia-Pacific region recording the lowest at 9.82 per 100,000. White non-Hispanic individuals exhibit the highest incidence rate, although the incidence among Black and Asian populations is also considerable. Clinical manifestations of PBC, such as cholestatic pruritus, abdominal discomfort, and fatigue, significantly impair patients’ quality of life (3).

Osteoporosis is a prevalent skeletal disorder marked by the deterioration of bone tissue microstructure and a decrease in bone mineral density (BMD) (4). This condition leads to reduced bone strength and increased fragility, consequently elevating the risk of fractures. Established risk factors for osteoporosis include aging, endocrine disorders, malnutrition, obesity, and the use of medications that impact bone metabolism. Osteoporosis-related fractures represent a substantial economic burden; for instance, annual expenditures amount to approximately $17.9 billion in the United States and £4 billion in the United Kingdom (5). Therefore, identifying the causes and risk factors for osteoporosis is crucial for early diagnosis and treatment, reducing fracture risk, and enhancing the quality of life.

A review study reported that the prevalence of osteoporosis in PBC ranged from 20% to 44%, with an increase in prevalence correlating to disease progression and a high incidence of fractures (10-20%) (6). A comprehensive analysis of 3,980 PBC patients revealed a significantly higher fracture risk and post-fracture mortality in these patients compared to matched controls from the general population (7). However, the relationship between PBC and osteoporosis is subject to debate. A retrospective study indicated that the susceptibility to osteoporosis in PBC patients was not higher than in the general population, with factors such as age and gender being more influential (8). Some researchers have also noted no significant difference in bone loss between PBC patients and healthy controls, suggesting that the risk of osteoporosis in PBC patients is more closely related to age and menopausal status than to the severity of liver disease (9).

The pathogenesis of osteoporosis in PBC is multifactorial, primarily involving reduced bone formation and increased bone resorption in the later stages of the disease. Studies suggest that elevated bile acids and bilirubin may contribute to osteoporosis by inducing apoptosis in osteoblasts and stimulating osteoclast activity (10). Osteoporosis can be considered an extrahepatic complication of PBC, with its etiology and pathogenesis still not fully understood. A study involving European and Chinese Han populations identified a genetic link between PBC and osteoporosis (11). The CLDN14 gene, encoding the tight junction protein claudin-14, plays a role in regulating epithelial cell tight junctions and bile secretion. A variant, rs170183, in CLDN14 has been linked to kidney stones and reduced BMD. Thus, the CLDN14 gene may serve as a potential molecular connection between PBC and osteoporosis, though its function and mechanism require further experimental validation and clarification. To date, no Mendelian randomization studies have explored the inherent causal relationship between these two conditions, leaving open the possibility of confounding or reverse causality. Further investigation into the causal relationships underlying these associations is warranted.

Traditional observational studies are often limited by confounding factors, reverse causality, and selection bias. Mendelian randomization (MR), utilizing genetic variants as instrumental variables (IVs) derived from genome-wide association studies (GWAS) data, offers a method to estimate the causal effect between exposure and outcome (12). Genetic variants, randomly assorted during meiosis and fixed at conception, act as long-term stable exposure factors unaffected by environmental, social, or other factors. This method thus overcomes the limitations inherent in conventional observational studies. In our study, we employed a bidirectional two-sample MR analysis to evaluate the causal relationship between PBC and osteoporosis.

## 2 Materials and methods

### 2.1 Data source

To investigate the causal relationship between PBC and osteoporosis, we selected single nucleotide polymorphisms (SNPs) as IVs from the GWAS database. This database is publicly accessible, eliminating the need for additional ethical approval. The PBC GWAS data were derived from a dataset published by Cordell et al. in the Journal of Hepatology (https://www.ebi.ac.uk/gwas/labs/publications/34033851) (13), representing one of the largest PBC GWAS datasets available. This study identified multiple genetic loci and genes associated with PBC. We included all five European cohorts, encompassing 8,021 PBC cases, 16,489 controls, and 5,186,747 SNPs. For PBC definition, these cohorts adhered to the criteria set by the European Association for the Study of the Liver (EASL) (3). The osteoporosis GWAS data were obtained from the FinnGen R9 database (https://www.finngen.fi/en), including 7,300 cases and 358,014 controls from the European population. The dataset employed ICD-10, ICD-9, and ICD-8 codes for osteoporosis diagnosis (14).

### 2.2 Genetic instrument selection

**Figure 1:**
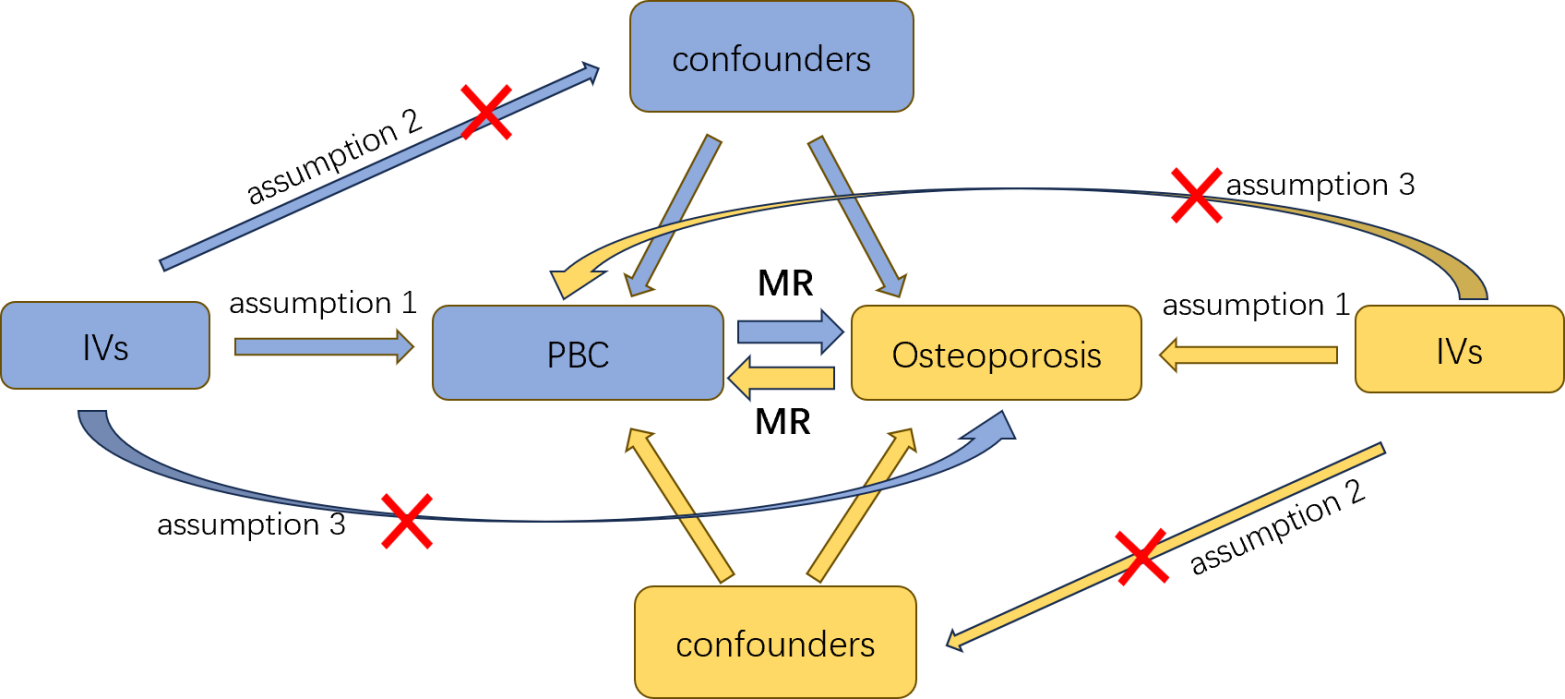
An overview of this Mendelian randomization (MR) study design; IVs, instrumental variables; MR, Mendelian randomization.

The selected genetic IVs had to fulfill three core assumptions of MR analysis to minimize result bias (15): 1. Relevance assumption: direct association with the exposure. 2. Independence assumption: independence from confounders of the exposure-outcome association. 3. Exclusion restriction assumption: influence on the outcome solely through the exposure (**Figure 1**). To mitigate potential interference from linkage disequilibrium between SNPs and ensure accurate and reliable causal inference for PBC and osteoporosis, we implemented various restrictive measures for SNP selection. We chose SNPs that were genome-wide and significantly associated with the exposure (*P*<5×10^-8^) and excluded those exhibiting high linkage disequilibrium (r^2^<0.001, kb<10,000) (16). To maintain the accuracy of the MR analysis, it was necessary to filter out palindromic SNPs, which are SNPs with effect alleles and other alleles as complements. Additionally, we evaluated the strength of the association between each IV and the exposure, excluding weak IVs. The F-statistic for each IV was calculated using the formula: F=Beta^2^/SE^2^ (17), where Beta represents the estimated effect of the allele on the exposure, and SE is the standard error. IVs with an F-statistic less than 10 were excluded due to potential genetic confounding or measurement error. Ultimately, we used these rigorously selected SNPs as the final IVs for subsequent Mendelian randomization analysis.

### 2.3 Statistical analysis

In our study, we employed five different methods for MR analysis to explore the causal relationship between PBC and osteoporosis: the inverse-variance weighted (IVW) method, MR-Egger, weighted median, weighted mode, and simple mode. The IVW method, which uses all valid SNPs, is advantageous for increasing statistical power. In the absence of heterogeneity and pleiotropy, IVW can provide the most accurate and efficient causal estimate, making it our primary method (18). The MR-Egger method introduces an intercept term to the IVW, enabling the detection and adjustment for horizontal pleiotropy. However, it may yield unstable or inaccurate estimates due to its susceptibility to outliers or unevenly distributed IVs (19). The weighted median offers better robustness and reduced finite-sample bias compared to the IVW but tends to have larger standard errors and lower efficiency (20). We also employed the simple mode and weighted mode methods to further validate the potential causality between exposure and outcome. A *P*-value threshold of <0.05 was applied in all MR analyses to ascertain the causal effects of exposure on the outcome.

### 2.4 Pleiotropy, heterogeneity, and sensitivity evaluation

Given that the IVW method cannot adequately address horizontal pleiotropy and confounding among SNPs, and may be influenced by outliers or influential SNPs, it was necessary to check for heterogeneity and pleiotropy before using IVW, and to exclude outliers or influential SNPs (18). We utilized Cochran’s Q test to examine heterogeneity among estimates from different genetic variants. A *P*-value of less than 0.05 indicated significant heterogeneity (21). The MR-PRESSO and MR-Egger’s intercept tests were used to provide valid MR estimates in the presence of horizontal pleiotropy. MR-PRESSO identifies genetic variants significantly impacting the causal estimate, eliminates the interference from outliers, and offers corrected results after their removal (22). MR-Egger’s intercept test serves to detect and estimate horizontal pleiotropy, and also provides a sensitivity analysis for the robustness of MR results (19). A leave-one-out analysis was conducted to evaluate the influence or bias of each SNP on the pooled estimate. All MR analyses and tests were performed using the “TwoSampleMR” and “MRPRESSO” packages in R software (version 4.3.1).

## 3 Result

### 3.1 MR analysis

We identified 47 SNPs significantly associated with the risk of PBC. These SNPs exhibited no linkage disequilibrium (r^2^<0.001) and were not considered weak instrumental variables, as their F-statistics all exceeded 10, fulfilling our previously established selection criteria. Nine of these SNPs did not have corresponding results in the osteoporosis-related GWAS database. Additionally, six palindromic SNPs were excluded. Consequently, we utilized the remaining 32 SNPs as IVs for MR analysis. To compute the proportion of phenotypic variance explained by the IVs, the following formula was used: R^2^ = [2 × Beta^2^ × (1 − EAF) × EAF]/[2 × Beta^2^ × (1 − EAF) × EAF + 2 × SE^2^ × N × (1 − EAF) × EAF]. Here, EAF is the effect allele frequency, N is the GWAS sample size of the exposure. The analysis revealed that these SNPs accounted for 14.55% of the PBC risk variance, indicating that the selected SNPs possessed strong predictive power and were capable of effectively minimizing confounding effects. Detailed information on these SNPs is presented in the appendix (**Supplementary Table S1**).

**Figure 2:**
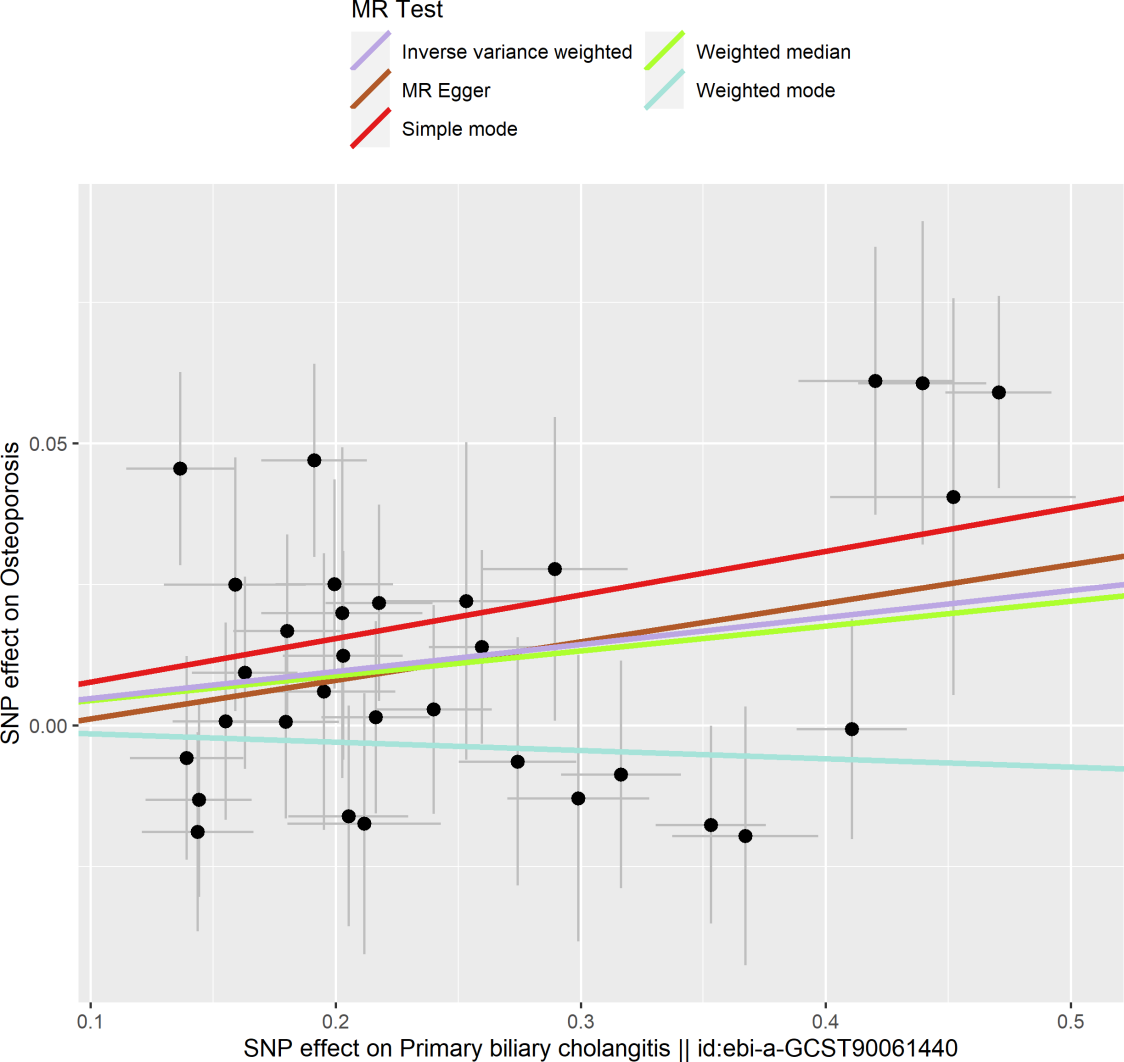
Scatter plot of the effect of primary biliary cholangitis on osteoporosis.

**Figure 3:**
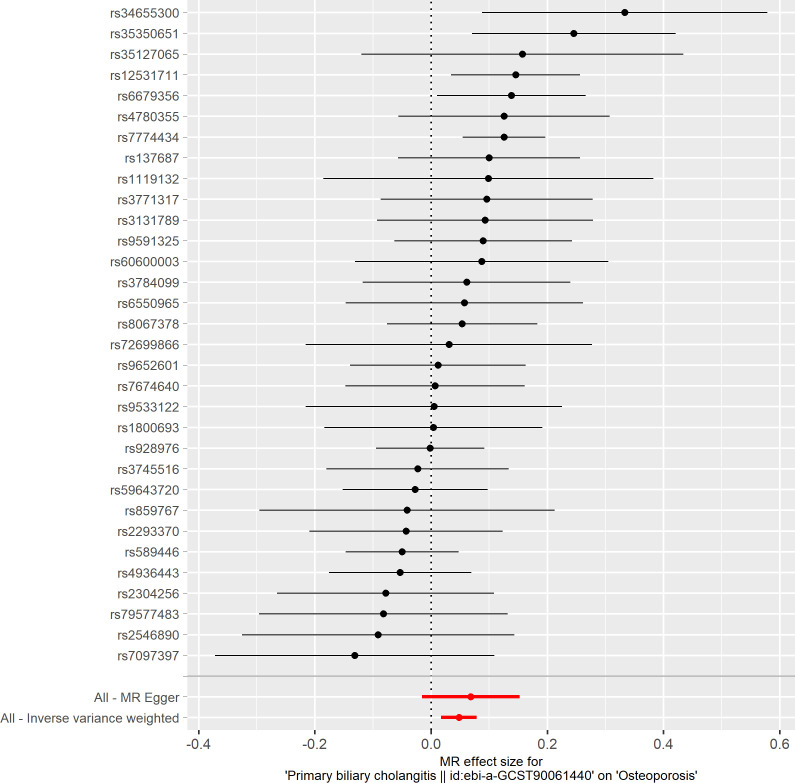
Forest plot of the effect of primary biliary cholangitis on osteoporosis.

**Figure 4:**
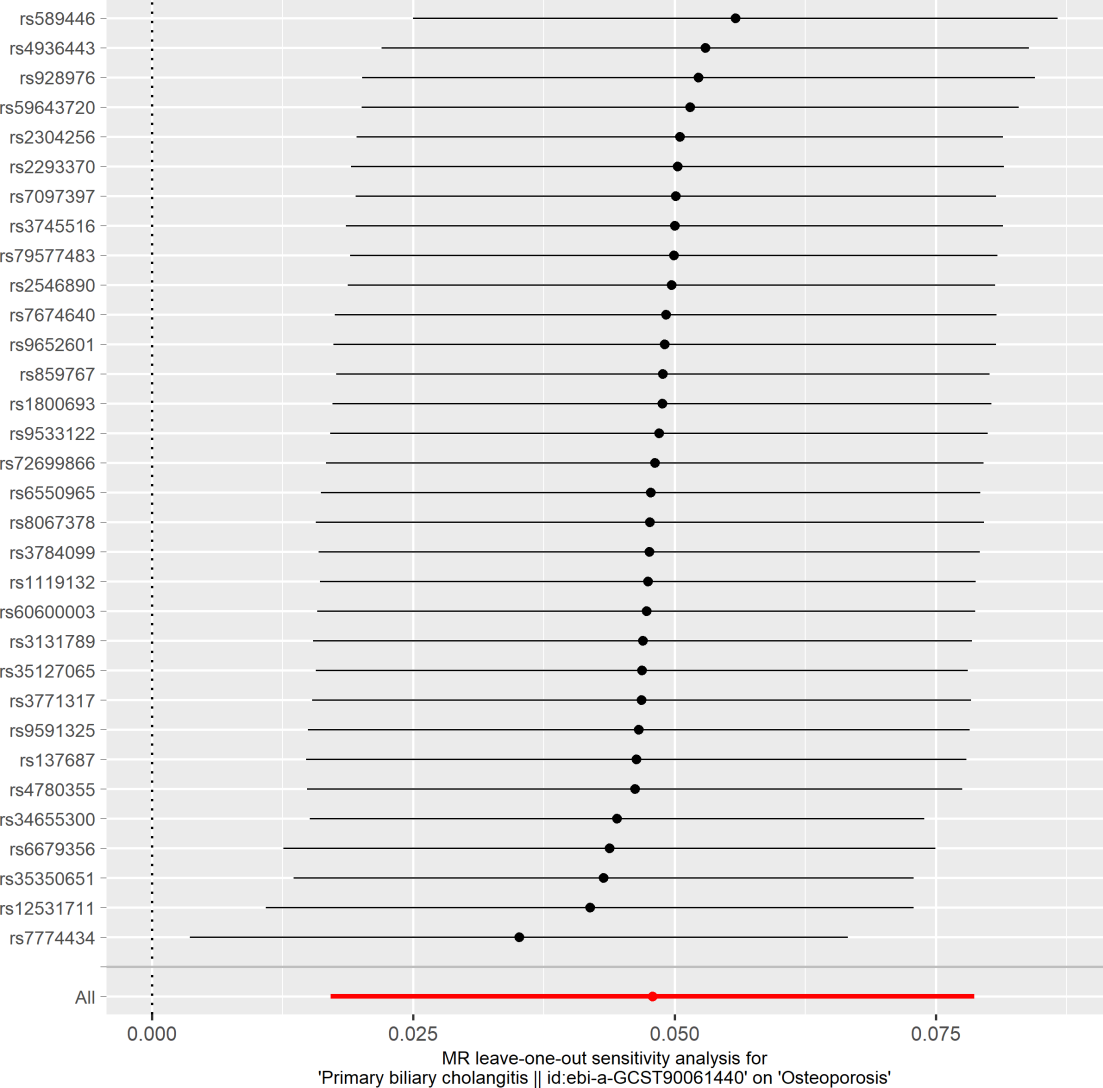
Leave-one-out plot of the effect of primary biliary cholangitis on osteoporosis.

**Figure 5:**
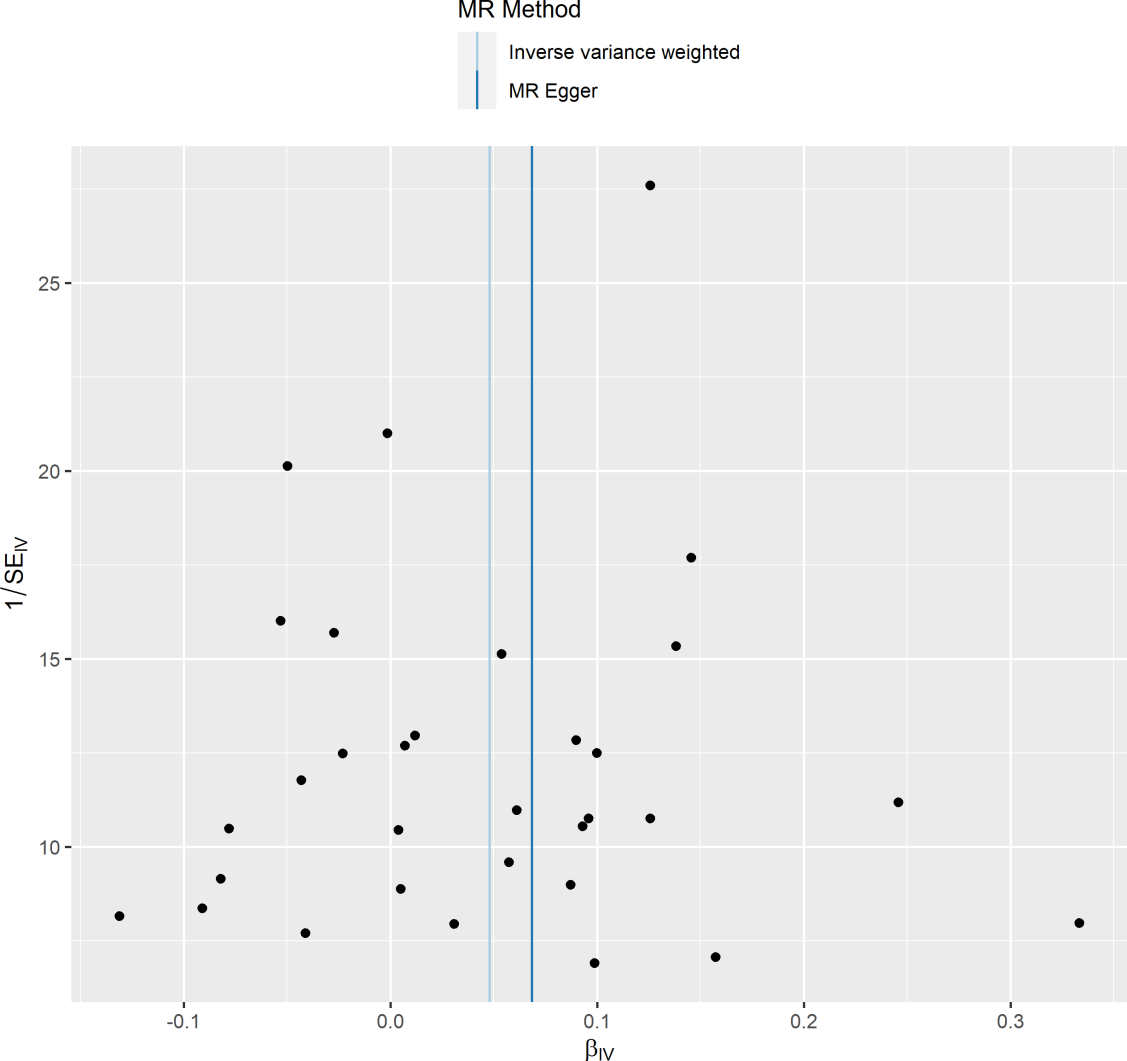
Funnel plot of the effect of primary biliary cholangitis on osteoporosis.

Based on the IVW method (OR: 1.049, 95%CI: 1.017–1.082, *P*=0.002), we found a statistically significant positive causal relationship between the risk of PBC and osteoporosis. The weighted median method also indicated a significant causal effect (OR: 1.045, 95%CI: 1.002–1.089, *P*=0.038). However, the MR-Egger method (OR: 1.071, 95%CI: 0.984–1.165, *P*=0.121) and the simple mode (OR: 1.080, 95%CI: 0.974–1.199, *P*=0.155) showed a directionally consistent but statistically non-significant positive causal relationship. The weighted mode (OR: 0.985, 95%CI: 0.872–1.114, *P*=0.815) yielded a non-significant effect estimate, diverging from the other methods’ results (**Table 1**). The weighted mode and simple mode are two distinct causal effect estimation methods in MR analysis. The weighted mode assigns weights based on the variance of the genetic instruments, whereas the simple mode does not consider variance. Typically, the simple mode’s accuracy is low, while the weighted mode can be inaccurate in small samples and is affected by the degree of association between genetic instruments and the exposure and outcome variables. In cases where the degree of association is inconsistent, the weighted mode may introduce bias, whereas the simple mode is more robust (23). In this study, the weighted mode’s results were the only ones inconsistent with the other methods and lacked statistical significance, potentially due to the influence of certain genetic instruments. Our primary findings are derived from the IVW method, which utilizes all valid genetic instruments, thereby enhancing statistical power and offering the most accurate and efficient results in the absence of heterogeneity and horizontal pleiotropy. Consequently, the results from the weighted mode, which diverged from the findings of other methods and lacked statistical significance, are deemed unreliable and do not significantly impact the overall conclusion of the Mendelian randomization analysis. Instead, more weight is given to the IVW method, widely considered the most robust and reliable among the available approaches. We conducted heterogeneity tests for all IVs, using Q statistics to evaluate the differences among them. The Q statistic was calculated to be 41.27 (*P*=0.103), indicating no significant heterogeneity. Additionally, the MR-PRESSO global test did not detect any outlier SNPs or horizontal pleiotropy effects of PBC on osteoporosis (*P*=0.156), and MR-Egger’s intercept test also found no evidence of horizontal pleiotropy (Egger intercept = -0.0057, *P*=0.610). These results suggest that the analysis is less likely to be influenced by potential confounding biases. A sensitivity analysis was conducted using the leave-one-out method, which involved sequentially removing SNPs, recalculating the causal effect with the remaining SNPs, and observing whether the results varied with each SNP removal. This analysis demonstrated stable results, further affirming the reliability of our findings (**Table 2**; **Figures 2**–**5**).

**Table 1:** Mendelian randomization estimates for PBC on osteoporosis.

Exposure	Outcome	No. of IVs	Methods	Beta	SE	OR (95%CI)	P
PBC	Osteoporosis	32	MR Egger	0.068	0.043	1.071 (0.984~1.165)	0.121
			Weighted median	0.044	0.021	1.045 (1.002~1.089)	0.038
			IVW	0.047	0.016	1.049 (1.017~1.082)	0.002
			Simple mode	0.077	0.053	1.080 (0.974~1.199)	0.155
			Weighted mode	-0.015	0.063	0.985 (0.872~1.114)	0.815

PBC, primary biliary cholangitis; IVs, instrumental variables; IVW, inverse variance weighting; SE, standard error; OR, odds ratio; CI, confidence interval.

P < 0.05 was considered statistically significant.

**Table 2:** Heterogeneity and horizontal pleiotropy for Mendelian randomization analysis.

Exposure	Outcome	Heterogeneity		Horizontal pleiotropy		
		Cochran’s Q	P	Egger intercept	SE	P
PBC	Osteoporosis	41.27	0.103	-0.0057	0.0110	0.610
Osteoporosis	PBC	1.434	0.921	0.1067	0.0994	0.342

PBC, primary biliary cholangitis.

P < 0.05 was considered statistically significant.

### 3.2 Further analyses

**Figure 6:**
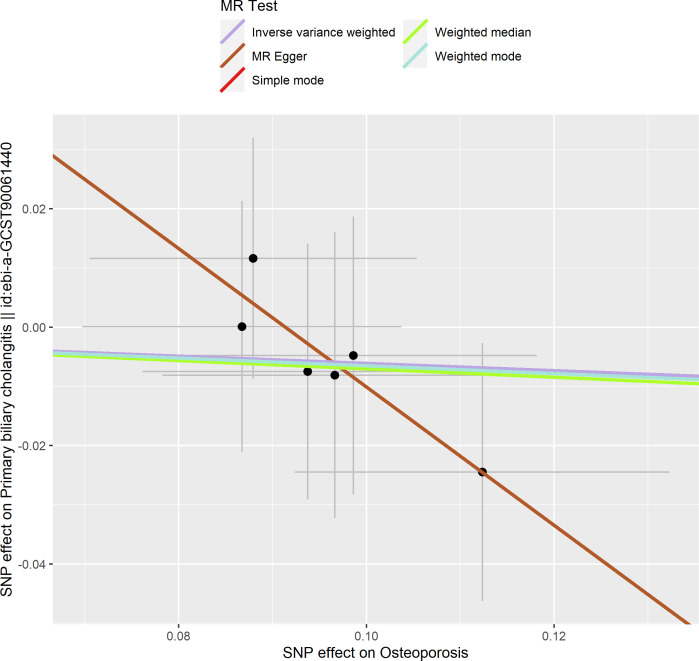
Scatter plot of the effect of osteoporosis on primary biliary cholangitis.

**Figure 7:**
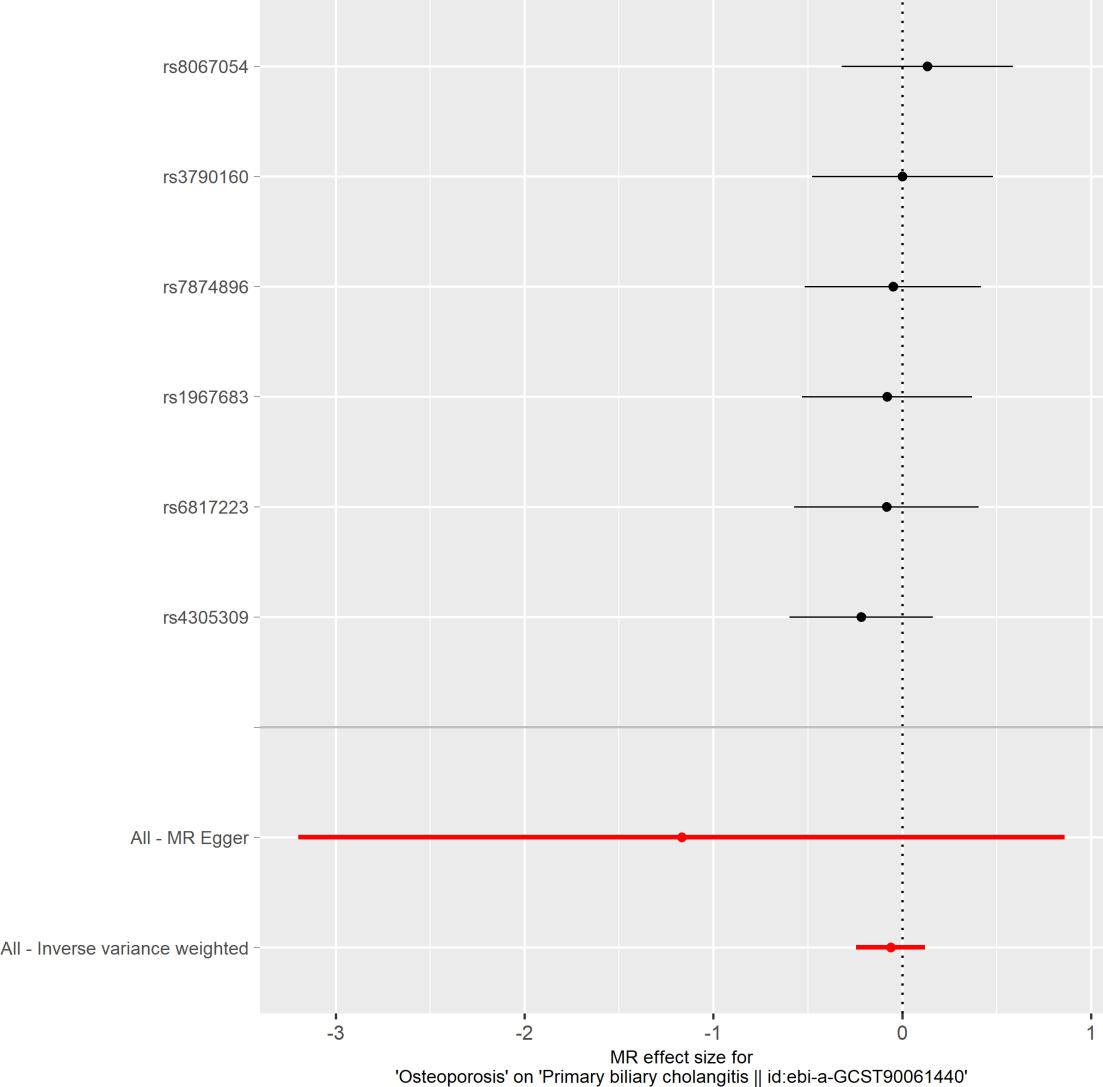
Forest plot of the effect of osteoporosis on primary biliary cholangitis.

**Figure 8:**
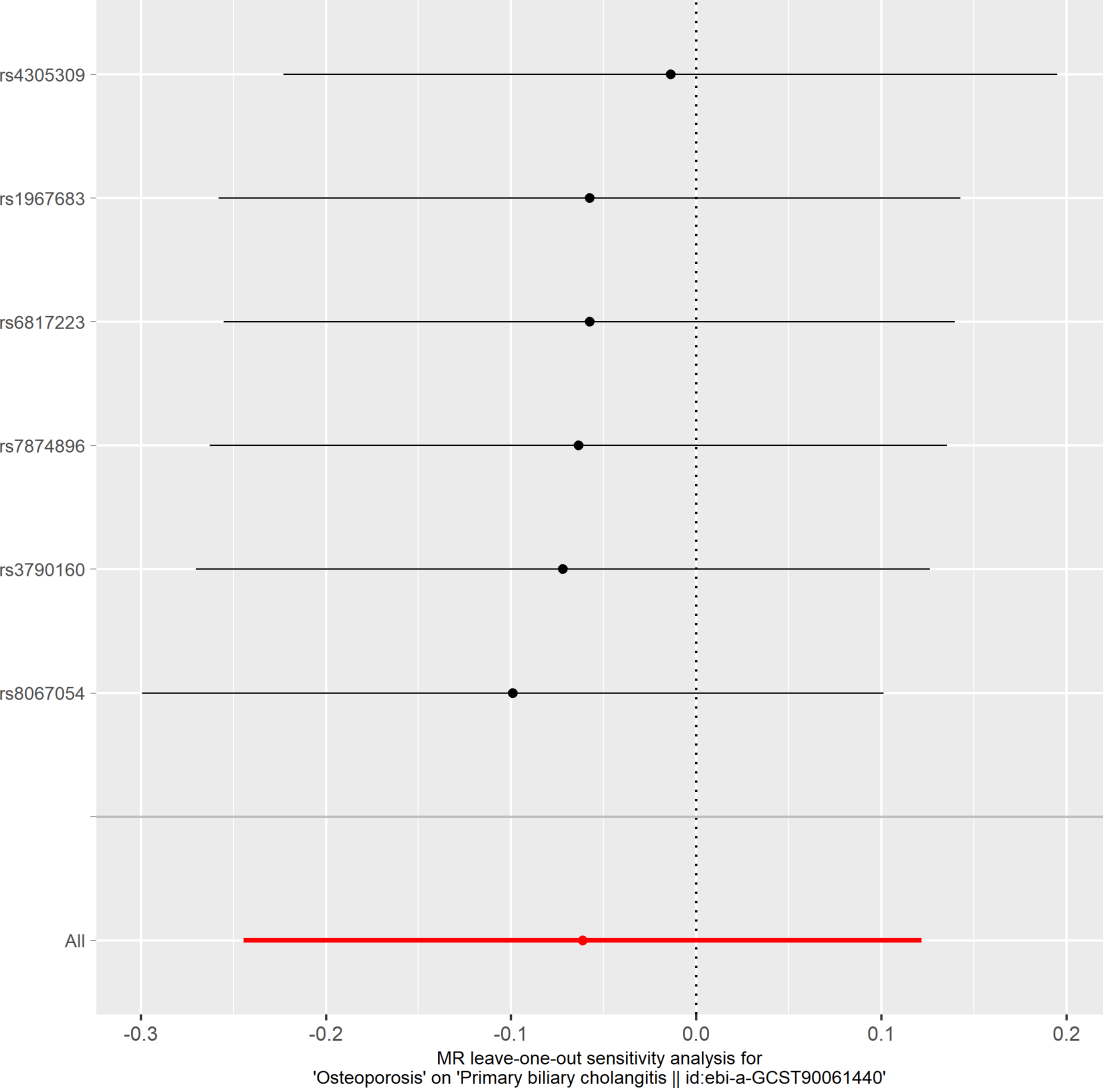
Leave-one-out plot of the effect of osteoporosis on primary biliary cholangitis.

**Figure 9:**
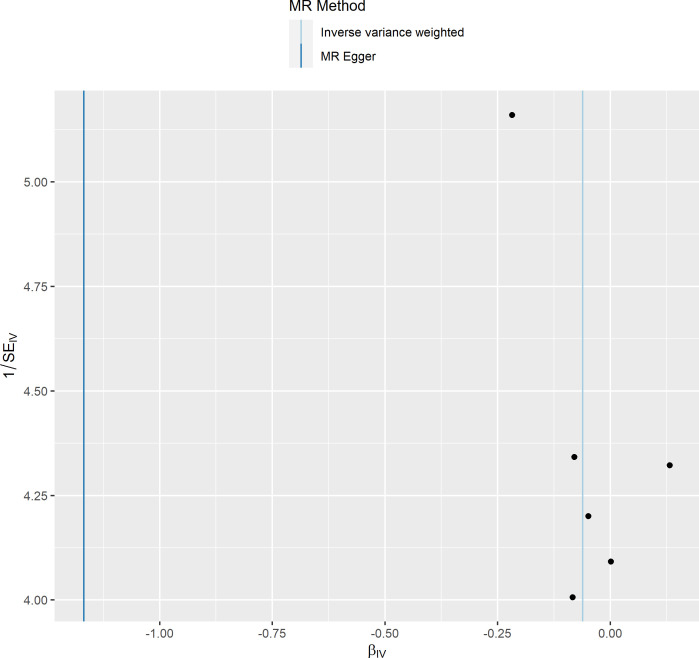
Funnel plot of the effect of osteoporosis on primary biliary cholangitis.

To delve deeper into the causal relationship between osteoporosis and PBC, we carried out a two-sample MR analysis with osteoporosis as the exposure and PBC as the outcome. We employed the same GWAS databases, IV selection methods, analysis techniques, and test procedures as previously described. When exposure-related SNPs were absent in the result dataset, we utilized alternative SNPs that demonstrated a high correlation with the relevant SNPs (r^2^>0.8). We loosened the relevance assumption and initially identified 14 SNPs significantly associated with disease risk from GWAS (*P*<5×10^-7^, r^2^<0.001). However, 8 of these SNPs were excluded because they lacked corresponding results in the PBC GWAS database, and no proxy SNPs were available. The F-statistics for the remaining SNPs were all above 10, indicating the absence of weak IV bias. Consequently, we used these 6 SNPs as IVs for our MR analysis (**Supplementary Table S2**).The results indicated no causal relationship between osteoporosis and the risk of PBC, as evidenced by the IVW analysis (OR: 0.941, 95%CI: 0.783–1.129, *P*=0.511), and similar conclusions were drawn from the other four methods (**Table 3**). Furthermore, we conducted heterogeneity tests on all IVs using the Q statistic to assess differences among them. The Q statistic was 1.434 (*P*=0.921), indicating no significant heterogeneity. MR-PRESSO global test did not detect any outlier SNPs or horizontal pleiotropic effects of PBC on osteoporosis (*P*=0.922), and MR-Egger’s intercept test also found no evidence of horizontal pleiotropy (Egger intercept = 0.1067, *P*=0.342). These findings suggest that our analysis is credible (**Table 2**; **Figures 6**–**9**).

**Table 3:** Mendelian randomization estimates for osteoporosis on PBC.

Exposure	Outcome	No. of IVs	Methods	Beta	SE	OR (95%CI)	P
Osteoporosis	PBC	6	MR Egger	-1.168	1.035	0.311 (0.041~2.363)	0.322
			Weighted median	-0.070	0.112	0.932 (0.748~1.161)	0.530
			IVW	-0.061	0.093	0.941 (0.783~1.129)	0.511
			Simple mode	-0.065	0.162	0.937 (0.682~1.287)	0.703
			Weighted mode	-1.168	1.035	0.311 (0.041~2.363)	0.322

PBC, primary biliary cholangitis; IVs, instrumental variables; IVW, inverse variance weighting; SE, standard error; OR, odds ratio; CI, confidence interval.

P < 0.05 was considered statistically significant.

## 4 Discussion

Primary biliary cholangitis (PBC) is a chronic cholestatic liver disease frequently associated with the development of osteoporosis. In our study, we employed bidirectional two-sample Mendelian randomization (MR) analysis to investigate the potential causal relationship between PBC and osteoporosis. This approach complements previous observational studies that have explored the linkage between these conditions. For instance, a study by Menon et al., published in the Journal of Hepatology in 2001, found a significant positive correlation between bone loss rate and bilirubin level in PBC patients. This finding suggests that the severity of liver disease is a crucial factor influencing the onset and progression of osteoporosis in these patients (24). Another study involving 176 PBC patients assessed the incidence, risk factors, and progression rate of osteoporosis, concluding that PBC is significantly associated with osteoporosis, with the severity of liver disease being an influential factor affecting the occurrence and progression of osteoporosis (25). These observational studies are consistent with the findings of our study.

Saeki et al. conducted research evaluating skeletal muscle disease in 117 PBC patients (26). Their findings revealed higher rates of osteoporosis, muscle atrophy, and vertebral fractures in this group, indicating a close interrelationship among these complications. Additionally, an article focusing on PBC and osteoporosis highlighted the strong connection between the two and analyzed various factors that may impact bone metabolism in PBC patients, such as vitamin deficiencies, IGF-1, BMP, bile acids, and bilirubin. This study concluded that individuals with PBC are more susceptible to osteoporosis and fractures than the general population, and the risk escalates as the disease progresses (6). In recent years, the exploration of the intestinal microbiota has gained prominence in research. PBC and osteoporosis share common intestinal microbial groups, including Candidatus_Soleaferrea, Eubacterium_coprostanoligenes_group, Allisonella, and Peptococcus. These microbes are significantly associated with both PBC and osteoporosis and may influence liver function, calcium absorption, intestinal barrier integrity, immune response, and bone density through various metabolic products or mechanisms (27). These studies collectively indicate that PBC is a systemic metabolic disease affecting not only the liver but also bone health. This is in alignment with the findings of our MR analysis.

The causal relationship between PBC and osteoporosis is underpinned by several hypotheses, reflecting the complexity of this association. Initially, the issue of osteoporosis in PBC patients was acknowledged and attributed to hepatic osteodystrophy, but further exploration was limited by the technological and hardware constraints of the time (28). One key factor in normal bone metabolism involves the interaction of a three-molecule complex comprising osteoprotegerin (OPG), nuclear factor-kB receptor activator ligand (RANKL), and RANK. It has been observed in some studies that the progression of PBC can reduce OPG secretion by the liver, leading to uncontrolled osteoclast activity and increased bone resorption. This is considered one of the potential mechanisms. However, there is also evidence suggesting that PBC-related osteoporosis primarily results from impaired bone formation, though excessive bone resorption might also play a role (29). Thus, the precise mechanism of osteoporosis linked to PBC remains not fully understood and likely varies depending on the severity of liver disease. Patients with PBC exhibit elevated levels of inflammatory cytokines, including IL-1, IL-6, TNF-α, which can directly or indirectly promote osteoclast formation and activation via the RANKL-RANK signaling pathway, leading to increased bone resorption and osteoporosis (30). Additionally, cholestasis in PBC patients raises levels of lithocholic acid and bilirubin, which can adversely affect osteoblasts and osteoblast-like cells, such as SAOS-2, through bile acid transport proteins like bile acid transport protein (BSEP) and multidrug resistance-associated protein (MRP). This impact results in reduced differentiation and mineralization, as well as increased apoptosis of these cells, contributing to osteoporosis (31). PBC also increases the risk of malabsorption and deficiencies of fat-soluble vitamins like A, D, E, and K, impacting bone metabolism and coagulation. Vitamin D is crucial for regulating bile acid metabolism and transport, inhibiting inflammation due to bile stasis, protecting bile duct cells, and suppressing liver fibrosis. Vitamin D deficiency, commonly observed in PBC patients, is associated with bone complications of the disease (32). Vitamin K acts as a coenzyme to activate bone formation proteins like osteocalcin (OC) and matrix Gla protein (MGP), maintaining bone stability and strength. A deficiency in Vitamin K can impair the function of OC and MGP, increasing the risk of osteoporosis and fractures (33). Moreover, estrogen, which inhibits osteoclasts, plays a role. PBC is more prevalent in middle-aged and older women, and post-menopausal decreases in estrogen levels can lead to excessive bone resorption and a heightened risk of osteoporosis (34).

Patients with PBC require comprehensive assessment and treatment strategies to improve their bone and muscle function and overall quality of life. The higher incidence and mortality of fractures observed in PBC patients underscore the need for early bone density testing to mitigate the risk of osteoporosis and fractures (10). Parés A, Guañabens N, et al. have also emphasized the importance of regular bone density monitoring and the supplementation of calcium and vitamin D in PBC patients (35). As PBC progresses, the associated bone loss intensifies, necessitating drug treatments that address both PBC and osteoporosis. Calcium and vitamin D supplements can be beneficial for bone health in PBC patients. However, the treatment of osteoporosis in this group is not well-explored, and the effectiveness of current medications remains uncertain. A systematic review and meta-analysis indicated that hormone replacement therapy and other drugs like calcitriol do not significantly reduce fracture risk or increase bone density in PBC patients (36), as the pathogenesis of PBC-related osteoporosis differs from that of postmenopausal osteoporosis, primarily stemming from reduced bone formation.

This study has limitations that should be addressed in future research. Firstly, it utilized GWAS data from European populations, limiting the generalizability of the results to other ethnicities or populations. Secondly, gender may significantly influence PBC and osteoporosis, as both conditions are more prevalent in middle-aged and elderly women. Due to the data sets’ limited sample size, this study did not stratify by gender and age, potentially introducing confounders such as gender and age. Future studies could address these issues with larger, more diverse sample sizes and stratification by gender and age. Despite these limitations, our study has notable strengths. Firstly, it is the first to apply MR analysis to investigate the bidirectional causality between PBC and osteoporosis. Secondly, the MR method design allows us to overcome the interference of confounding factors on results and reverse causality, which are common issues in observational studies. Our sensitivity analysis further ensures the consistency and robustness of the causal estimates and results.

The future direction for treating osteoporosis induced by PBC remains an area of active exploration, highlighting the need for drugs that can both manage PBC and protect bone health. Denosumab, a medication that blocks the binding of RANKL to RANK and inhibits osteoclasts, thereby reducing bone resorption and increasing bone strength, shows potential (37). Intriguingly, some researchers have noted high expression of RANK in the bile duct cells of PBC patients, implicating the RANKL-RANK axis in the disease’s pathogenesis. This suggests that Denosumab might not only improve bone health but also prevent the progression of PBC and protect liver function (38). Another promising drug is abaloparatide, a synthetic peptide analog of parathyroid hormone-related protein. It is used for postmenopausal osteoporosis and has demonstrated superior efficacy and safety compared to teriparatide (39). Denosumab and abaloparatide may therefore be effective options for treating osteoporosis associated with PBC, but their impacts in this specific context are yet to be fully assessed and require further clinical trials for validation. Additionally, the development of new bone-forming medications tailored to PBC-related osteoporosis should be a focus of future research.

## 5 Conclusion

Our study concludes that PBC is a positive causal factor for the risk of osteoporosis. This association may be attributed to chronic inflammation, bile acid metabolism disorders, and vitamin D deficiency commonly seen in PBC patients. However, we did not establish a clear causal link between osteoporosis and the risk of developing PBC, indicating that osteoporosis does not appear to be a risk factor or early marker for PBC. These findings offer new perspectives and guidelines for managing the bone health of patients with PBC. We recommend regular bone density testing, calcium and vitamin D supplementation, and appropriate physical activity for these patients.

## Data availability statement

The datasets presented in this study can be found in online repositories. The names of the repository/repositories and accession number(s) can be found in the article/**Supplementary Material**.

## Author contributions

DZ: Data curation, Methodology, Software, Writing – original draft. GL: Validation, Writing – review & editing. WB: Conceptualization, Data curation, Investigation, Methodology, Software, Supervision, Validation, Writing – original draft, Writing – review & editing. JT: Conceptualization, Data curation, Investigation, Project administration, Supervision, Validation, Writing – review & editing. BY: Methodology, Software, Supervision, Writing – original draft, Writing – review & editing. CH: Conceptualization, Data curation, Formal Analysis, Investigation, Methodology, Project administration, Software, Supervision, Validation, Writing – review & editing.
