# Supplementary material for: Primary biliary cirrhosis and osteoporosis: a bidirectional two-sample Mendelian randomization study
Source: Front Immunol. 2023 Dec 14;14:1269069. doi: 10.3389/fimmu.2023.1269069 (PMC10755900; doi:10.3389/fimmu.2023.1269069)
Supplement: Supplementary file 1 [file DataSheet_1.pdf]

Supplementary Materials

Table S1. Detailed information of instrumental variables utilized in the MR analysis of the causal effects of PBC on osteoporosis risk.

SNP	Effect allele	Other allele	Beta	Se	P	R^2	F
rs1119132	G	A	0.20264	0.0328117	6.58E-10	0.001553725	38.14107148
rs12531711	G	A	0.4202	0.0313452	8.57E-41	0.007278704	179.7090737
rs137687	A	G	-0.217763	0.0218724	2.37E-23	0.004027904	99.12319741
rs1800693	C	T	0.179759	0.021699	1.19E-16	0.002792185	68.62807974
rs2293370	A	G	-0.299009	0.0289393	5.54E-25	0.004336723	106.7560453
rs2304256	A	C	-0.205245	0.0244295	4.43E-17	0.002871599	70.5855845
rs2546890	G	A	-0.144233	0.0216142	2.50E-11	0.001813507	44.52982025
rs3131789	G	A	0.180174	0.0219123	2.00E-16	0.002750861	67.60959124
rs34655300	T	C	0.136669	0.0220009	5.23E-10	0.001571928	38.58861037
rs35127065	T	C	0.159067	0.0289275	3.82E-08	0.001232139	30.23697746
rs35350651	AC	A	-0.191253	0.0214806	5.50E-19	0.003223874	79.27270842
rs3745516	G	A	-0.274319	0.0239747	2.65E-30	0.005313106	130.9198246
rs3771317	C	T	0.289503	0.0297719	2.40E-22	0.003843063	94.55685824
rs3784099	A	G	-0.203016	0.0243697	8.31E-17	0.002823507	69.4001114
rs4780355	C	T	-0.199551	0.0240186	1.01E-16	0.002808328	69.0259572
rs4936443	T	C	0.367095	0.0297432	5.39E-35	0.006176572	152.3286368
rs589446	T	G	-0.353093	0.0224396	1.96E-55	0.010000909	247.598491
rs59643720	C	A	0.316412	0.0244524	2.73E-38	0.006785202	167.441424
rs60600003	G	T	0.253264	0.035012	4.70E-13	0.002130314	52.32546349
rs6550965	A	C	0.163094	0.0215365	3.65E-14	0.002334358	57.3489826
rs6679356	T	C	-0.439362	0.0261434	6.61E-63	0.011392044	282.4365185
rs7097397	A	G	-0.143772	0.0227055	2.42E-10	0.001633177	40.0946557

rs72699866	A	G	-0.195158	0.0293378	2.89E-11	0.00180215	44.25044376
rs7674640	T	C	0.216421	0.0220898	1.56E-22	0.003900986	95.9876217
rs7774434	C	T	0.470454	0.0216556	3.68E-104	0.01889155	471.9477163
rs79577483	G	A	0.211743	0.031246	1.23E-11	0.001870135	45.92289591
rs8067378	G	A	0.259633	0.0215258	1.75E-33	0.005900485	145.4792929
rs859767	G	A	-0.139305	0.0230646	1.54E-09	0.001486115	36.47888495
rs928976	T	C	0.410549	0.022469	1.50E-74	0.013438273	333.8585546
rs9533122	G	A	0.155125	0.021532	5.83E-13	0.002113162	51.90328514
rs9591325	C	T	-0.451884	0.050169	2.14E-19	0.003299169	81.13029214
rs9652601	A	G	-0.239954	0.0237919	6.69E-24	0.004132909	101.7179822

MR, Mendelian Randomization; PBC, Primary biliary cirrhosis; SNP, single nucleotide polymorphism; SE, standard error.

Table S2. Detailed information of instrumental variables utilized in the MR analysis of the causal effects of osteoporosis on PBC risk.

SNP	Effect allele	Other allele	Beta	Se	P	R^2	F
rs1967683	C	T	-0.09375	0.0176141	1.02E-07	7.75393E-05	28.32837335
rs3790160	C	T	0.0867476	0.0170103	3.40E-07	7.11859E-05	26.00704388
rs4305309	C	T	0.112353	0.0199401	1.76E-08	8.68982E-05	31.74787652
rs6817223	C	T	0.0966298	0.0183851	1.47E-07	7.56121E-05	27.62422918
rs7874896	T	G	-0.0986055	0.0195676	4.67E-07	6.95073E-05	25.39376846
rs8067054	T	C	-0.0879418	0.0174264	4.50E-07	6.97074E-05	25.46685668

MR, Mendelian Randomization; PBC, Primary biliary cirrhosis; SNP, single nucleotide polymorphism; SE, standard error.
